# Empirical Study on the Sustainability of China’s Grain Quality Improvement: The Role of Transportation, Labor, and Agricultural Machinery

**DOI:** 10.3390/ijerph15020271

**Published:** 2018-02-05

**Authors:** Ming Zhang, Fang Duan, Zisen Mao

**Affiliations:** 1 School of Political Science and Public Administration, Southwest University, Chongqing 400715, China; zhming523@swu.edu.cn; 2 School of Business Administration, Shanghai Lixin University of Accounting and Finance, Shanghai 201620, China; 3 Department of Mathematics, The Army Engineering University of PLA, Nanjing 211101, China; maozisen@126.com

**Keywords:** grain quality improvement, stochastic frontier model, quality improvement, quality improvement efficiency

## Abstract

As a major part of farming sustainability, the issues of grain production and its quality improvement have been important in many countries. This paper aims to address these issues in China. Based on the data from the main production provinces and by applying the stochastic frontier analysis methodology, we find that the improvement of transportation and the use of agricultural machinery have become the main driving forces for grain quality improvement in China. After further studying different provinces’ potentials of grain quality improvement, we show that grain quality has increased steadily. Therefore, we can conclude China’s grain quality improvement is indeed sustainable. Furthermore, different grains like rice, wheat, and corn share similar characteristics in terms of quality improvement, but the improvement rate for rice is relatively low, while those of corn and wheat are relatively high. Moreover, the overall change of efficiency gain of grain quality improvement is not significant for different provinces. The efficiency gains of the quality improvements for rice and wheat even decrease slightly. In addition, we find that only expanding grain quality improvement potential can simultaneously achieve the dual objectives of improving grain quality and increasing yield.

## 1. Introduction

Grain is critical for human nutrition and public health, and there are many studies on how to improve grain yield. A lot of scholars analyzed the influencing factors of grain yield, such as agricultural machinery, land area and soil quality. Soliman and Ewaida [1] based on Egypt and van Zyl et al. [2] based on South Africa found that the use of agricultural machinery led to a marked increase in labor productivity in food production and an increase in output. Using Nigeria as a sample, Takeshima et al. [3] found that farmers using mechanical services can release more labor force to engage in non-farming activities. Other scholars found that land was an important factor affecting grain production, such as Deininger et al. [4] based on Indian and Lorenzetti [5] based on Switzerland. More research articles paid attention to soil quality and concluded that soil quality is very important to sustainable development [6,7]. Soil quality’s decline was found by many studies [8]. Also, the use of machinery was regarded as an important factor [9]. Lots of research emphasized the role of Controlled Traffic Farming [10], Soil and Water Conservation [11], and Straw Mulches [12] for reducing soil erosion.

Since the Reforming and Opening-Up, although China’s grain yield has experienced fluctuations, but the basic trend is rather positive, especially after the year of 2004. For 12 years from 2004 to 2015, the grain yield in China increased continuously from 4.96 million tons to 6.21 million tons [13]. Specifically, major grains such as rice, wheat, and corn have experienced a period of yield increase. The increase of grain yield or the improvement of the grain industry in general contribute greatly to grain security and is a fundamental basis for China to implement sustainable development [14]. A number of research studies focus on how to increase grain yield. To investigate the sustainability of grain yield improvement, many scholars analyzed from the perspective of the grain input factors. Ma and Li [15] found that the sowing area in China decreased from 1995 to 2005, and they contended that there were still some difficult problems in sustainable grain yield increase. Qu et al. [16] and other scholars argued that it would be difficult for China to make grain yield increase sustainable because even though grain yield per unit was an essential factor in grain yield, the sowing area was imposing a stronger and stronger constraint. Dong [17], however, analyzed from the perspective of farmers and used a questionnaire to study the problems of increasing the yields of three main crops in China. The results showed that technology, changing climate, and the quality of soil would become the main constraints. Only through innovation in technology can we guarantee the security of grain yield increase. Gao et al. [18] pointed out that China’s grain yield increase still had great potential. This can be done through expanding sowing areas and increasing grain yield per unit area. Long and Pu [19] argued that we could still effectively carry out measures to realize grain yield increase. However, to this end, we must keep stabilizing, strengthening, and perfecting the related subsidy policies. Based on the data of grain, the quantity of crops and the sowing area from 2003 to 2011, Liu et al. [20] studied the main factors contributing to China’s grain yield improvement since 2003 by applying the decomposition method of contribution factors. They found that from 2003 to 2011, China’s grain yield mostly depended on the denotative production mode that gave priority to increasing farming area. Because of the constraints of the grain consumption structure and international grain trade capacity, the future potential of structural and significant improvement was very small and the pressure for future grain yield increase would be higher and higher.

However, studies based on the perspective of inputs of China’s grain yield increase may be incomplete. To better study the sustainability of grain yield increase potential in China, analyzing both inputs and outputs is important. The analysis of the potential can be derived from studying technological progress. The maximum output from the production function where technology and input are fixed is called the production frontier or production potential [21]. As for the production frontier, the stochastic frontier analysis parametric model and nonparametric methods are both involved. Scholars all over the world have started to use stochastic frontier analysis parametric model and nonparametric methods to analyze the sustainability issues. However, as far as the China’s grain yield problem is concerned, these two methods still lack pertinence. When analyzing China’s grain yield problem by applying stochastic frontier analysis parametric model and nonparametric methods, Kalirajan et al. [22], Xu and Jeffrey [23], Chen and Huffman [24], and other scholars paid more attention to comparing agricultural production efficiencies in different time periods and different products. Chinese scholars, however, tend to focus more on analyzing technology efficiency. For instance, Qiao [25], Kang and Liu [26], Li et al. [27], Fan et al. [28], Huang and Zhou [29,30], Gao and Song [31], Gao and Ma [32], Tang and Vila [33], and Yang et al. [34] all assess the efficiency of China’s grain yield technology based on whether stochastic frontier model contains efficiency or not. In fact, as the stochastic frontier analysis methodology develops, especially the maturity of frontier technique of Battese and Coelli [35,36] with panel data, estimating the sustainability of production potential with stochastic frontier analysis technique has become more important. Wu [37] is the first one to use this technique to analyze the sustainability of Chinese economy. More recently, Shi and Li [21], Lu and Zhao [38], and He [39] have all successfully applied the stochastic frontier model.

This research aims to apply stochastic frontier model to the analysis of grain quality improvement. There is no doubt that the grain yield in China has enjoyed rapid growth, but the quality improvement related to Chinese grain is relatively slow. If we use the first-class rate as grain quality measure indicator, it is clear that rice quality is stable, but the quality of wheat and corn shows a slow downward trend [40]. According to the research conducted by Chinese Center for Disease Control and Prevention, the main problems of grain quality and safety lie in mycotoxin, residue of pesticide, and heavy metals in excess of the standards [41]. As a matter of fact, with the increased income of residents, their demand for grain quality is increasing. The poor quality of Chinese grain makes it less competitive when compared with high quality agricultural products from other countries [42]. This is a prominent problem for the development of the Chinese grain industry in the future [43]. To address this critical problem, at the beginning of 2014, the Chinese government put forward the idea of more emphasis on grain quality and safety. In the following years, it seems that the Chinese government will focus more on how to improve grain quality while maintaining its yield [44].

This research refers to the model in Wu [37] and introduces the stochastic frontier analysis framework to study the potential on grain quality improvement. Based on the panel data from 13 main grain producing areas from 2008 to 2015, and by analyzing the quality improvement potentials in these areas, our research investigates the sustainability of China’s grain quality improvement. The concept of sustainability in our paper is close to Shi and Li [21], which is the capability of steady grain quality improvement without harming the environment. Our research goal includes two. On the one hand, based on the stochastic frontier model, the paper builds the influencing factors of grain quality and the potential measurement model to investigate the dynamic change trend of the grain quality improvement potential. On the other hand, the paper estimates the relationship between grain quality and grain yield.

The important contributions of this paper can be summarized as follow. First, previous studies focus more on grain yield when analyzing China’s food security issues, while our study focuses on the quality issues in grain production using the stochastic frontier model. Second, our study evaluates the grain quality improvement potential in China based on a stochastic frontier model, which confirms the sustainability of grain quality improvement in China. Third, our study examines the relationship between grain quality and grain yield, which is a first in the literature.

## 2. Theoretical Analysis and Measurement Model of Grain Quality Improvement Potential

### 2.1. Theoretical Analysis of Grain Quality Improvement Potential

Production potential refers to the maximal output an economy entity can achieve when technique and input are fixed. Undoubtedly, the actual output is often not the maximal due to factors like inefficiency. Therefore, a gap often occurs between the actual output and the production potential, which represents efficiency loss to some extent. To measure production potential and its loss, the most common method is to obtain the fitted values based on the estimation from the regression model, use the fitted values as production potential, and compare them with the actual numbers to study the potential for increasing production [35,36]. According to this method, the fitted values from regression are actually average values closest to the actual values. The potential of grain quality improvement means the best quality when the grain yield input is fixed. It is an optimal value and the upper limit of actual value [37]. Thus, the meaning of the fitted values from the regression model is actually in conflict with the concept of quality-improvement potential [21].

**Figure 1:**
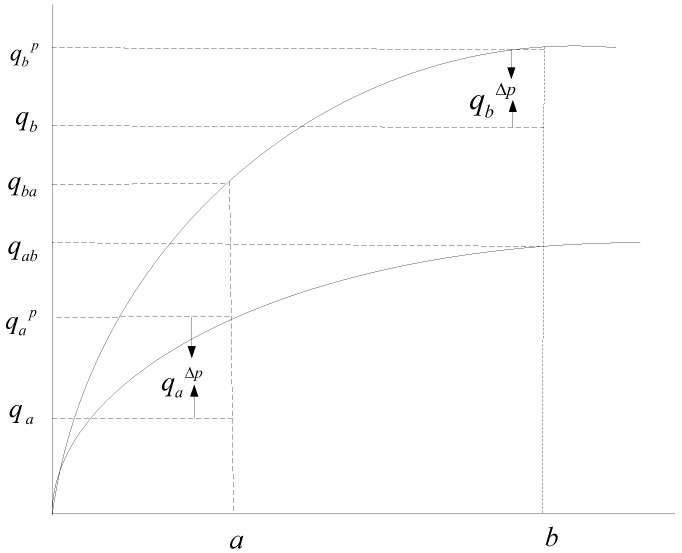
Model of grain quality improvement potential.

This article adopts the structure of economic yield-potential in different regions of China proposed first in Wu [37], which introduces the stochastic frontier model to the analysis of the potential of grain quality improvement. In a given quality function, we can estimate the potential value of grain quality. Using it as a basis, we can then analyze the potential of grain quality improvement in different regions in China. The specific analysis structure is shown in Figure 1.

In Figure 1, q represents grain quality, $q^{p}$ represents grain quality improvement potential, $q^{\Delta p}$ represents grain quality improvement efficiency. $q_{ab}$ refers to the maximum grain quality which the input b can reach in the frontier production function of a. $q_{ba}$ refers to the maximum grain quality which the input a can reach in the frontier production function of b. $q_{b}-q_{a}$ refers to grain quality gap between b and a. According to Figure 1, it can be written as below:

(1) $$q_{b}-q_{a}=(q_{b}{}^{p}-q_{b}{}^{\Delta p})-(q_{a}{}^{p}-q_{a}{}^{\Delta p})=(q_{b}{}^{p}-q_{ab})+(q_{ab}-q_{a}{}^{p})-(q_{b}{}^{\Delta p}-q_{a}{}^{\Delta p})$$

According to the function above, the change of grain quality can be divided into three parts. The first part $q_{b}{}^{p}-q_{ab}$ refers to the growth of quality improvement potential, which also means the increase of output when the input is fixed. As stated in Wu [37], this kind of grain quality improvement is sustainable. The second part $q_{ab}-q_{a}{}^{p}$ refers to the increase in quality caused by the increase of input when the frontier production function is fixed. This kind of increase is unsustainable. The third part $q_{b}{}^{\Delta p}-q_{a}{}^{\Delta p}$ refers to the efficiency gap which represents the gap of the capacity of two sides to realize quality improvement potential. In this way, with the help of frontier estimation techniques, the grain quality improvement can be divided into the change of quality improvement potential, the change of input factors, and the change of quality improvement efficiency.

Next, we need to construct a function to measure quality improvement potential. The paper uses a production function to implement it. In microeconomic theory, a production function is defined in terms of the maximum output that can be produced from a specified set of inputs, given the existing technology available [35,36]. It is similar to the concept of potential mentioned earlier. Lots of scholars believe that the econometric modelling of frontier production functions can provide useful insights into best-practice technology and measures of productive capability. Hence, this paper measures the change of quality improvement potential, the change of input factors and the change of quality improvement efficiency based on the stochastic frontier model.

Among these, the potential of grain quality improvement mainly depends on the development of technology. As Figure 1 shows, quality improvement potential refers to the increase of grain quality when the input is fixed, so it depends on the development of technology when other factors are fixed. The development of technology will improve quality. Due to the different levels of technology development in different regions, the potential of grain quality improvement also illustrates some provincial differences. Figure 1 also shows that most of the time, grain quality fails to reach the best quality, which means that efficiency loss exists. As there are many factors that affect efficiency, we will simplify it as the function of time in the analysis according to Wu [37] and Shi and Li [21].

### 2.2. Introduction of the Influencing Factors of Grain Quality and the Potential Measurement Model

According to the stochastic frontier model, not all producers are located at the frontier of the production function. Instead, there is a gap between the efficiency of most producers and the optimal efficiency. In other words, there is inefficiency. The relationship between actual quality, leading-edge quality, and efficiency can be represented by the following equation:

(2) $$q_{it}=f(x_{it},t)\exp(-\mu_{it})$$

In the above equation, i represents decision-making unit, t represents time, $q_{it}$ represents the actual quality of the t year in unit i, f() represents the determinate leading-edge quality in the stochastic frontier analysis function, and $x_{it}$ represents the vector of the input factors. $\exp(-\mu_{it})$ reflects efficiency loss, $\mu_{it}$ represents non-efficiency index, namely the relatively leading-edge efficiency level. Taking the log of both sides of Equation (2), we acquire the following equation:

(3) $$\ln q_{it}=\ln f(x_{it},t)-\mu_{it}$$

and derive the leading-edge item $\ln f(x_{it},t)$ of the time t

(4) $$\begin{array}{ll} \frac{d(\ln f(x_{it},t))}{dt} & =\frac{\partial (\ln f(x_{it},t))}{\partial t}+\sum_{i}\frac{\partial (\ln f(x_{it},t))}{\partial x_{i}}\times \frac{\partial x_{i}}{\partial t}\\ & =\frac{\partial (\ln f(x_{it},t))}{\partial t}+\sum_{i}\frac{\partial (\ln f(x_{it},t))}{\partial x_{i}/x_{i}}\times \frac{\partial x_{i}/x_{i}}{\partial t} \end{array}$$

In the above equations, $\frac{\partial (\ln f(x_{it},t))}{\partial t}$ represents the potential of growth, namely the change in quality with time when the input factors remain the same. The second item of the right side of the above equation measures the changes due to the increase in leading-edge quality function. $\frac{\partial (\ln f(x_{it},t))}{\partial x_{i}/x_{i}}$ represents the quality elasticity of factor $x_{i}$.$\frac{\partial x_{i}/x_{i}}{\partial t}$ represents the factor change rate.

Based on the stochastic frontier model and the framework of Wu [37], the paper introduced the time factor and input variable into the model and constructed the following determinant model of the grain quality

(5) $$\ln Q_{it}=c+\alpha_{1}t+\alpha_{2}t^{2}+\alpha_{3}\ln T_{it}+\alpha_{4}\ln L_{it}+\alpha_{5}\ln K_{it}+\alpha_{6}\ln F_{it}+\alpha_{7}\ln Z_{it}\;+\delta_{D}t\ln T_{it}+\delta_{L}t\ln L_{it}+\delta_{K}t\ln K_{it}+\delta_{F}t\ln F_{it}+\delta_{Z}t\ln Z_{it}+V_{it}-u_{it}$$

In the above equation, i represents region, t represents time, Q represents grain quality, T represents transportation, L represents the labor input, and K, F, and Z represents the number of agricultural machinery, fertilizer, and government funding put in the grain yield, respectively. $c+\alpha_{1}t+\alpha_{2}t^{2}$ represents the impact of national factors on grain quality improvement, especially neutral technological progress. $\alpha_{3}\ln T_{it}+\alpha_{4}\ln L_{it}+\alpha_{5}\ln K_{it}+\alpha_{6}\ln F_{it}+\alpha_{7}\ln Z_{it}$ represents the impact of factors on grain quality improvement. $\delta_{D}t\ln T_{it}+\delta_{L}t\ln L_{it}+\delta_{K}t\ln K_{it}+\delta_{F}t\ln F_{it}+\delta_{Z}t\ln Z_{it}$ represents the contribution of input-factor-biased technological progress to grain quality improvement as the time goes on. $V_{it}$ represents the stochastic disturbance, and $V_{it}$~N (0,$\sigma^{2}$); $u_{it}$ represents efficiency loss, with an assumption of obedience.

(6) $$u_{it}=u_{i}\exp[-\eta (t-N)]$$

In the above equation, $u_{i}$ was assumed to be non-negative truncations of the *N* (0,$\sigma_{u}{}^{2}$) distribution (i.e., half-normal distribution) or have exponential distribution. *η* represents the trend of the quality improvement efficiency as the time goes on. Obviously, if *η* > 0, efficiency will increase, and if *η* < 0, efficiency will decrease.

Based on Equation (5), the potential of grain quality improvement can be written as the following:

(7) gpotential=∂lnQ∂t=α1+2α2t+δDlnTit+δLlnLit+δKlnKit+δFlnFit+δZlnZit

Quality improvement efficiency can also be acquired through Equation (5), namely $TE_{it}=\exp(-u_{it})$. Assuming $u_{it}=u_{i}\times \exp[-\eta (t-N)]$, we can express the changes in leading-edge efficiency as

(8) $$TE_{it}=\exp(u_{i}\exp[-\eta (t-N)])$$

Based on (7) and (8), we can acquire grain quality improvement potential and efficiency and observe the potential of grain quality improvement within the test period and the features of dynamic changes in quality improvement efficiency. In this way, we can identify the driving factors of grain quality improvement in the same period and further estimate the sustainability of grain quality improvement.

## 3. Empirical Analysis on the Grain Quality Improvement Potential

For the empirical analysis, we employ China’s provincial panel data. To identify the driving forces in grain quality improvement, the sample period was set from 2008 to 2015. The sample contains 13 major grain producing provinces in China’s mainland, including Hebei, Henan, Heilongjiang, Jilin, Liaoning, Hubei, Hunan, Jiangsu, Jiangxi, Inner Mongolia, Shandong, Sichuan, and Anhui. These major grain-producing areas are the most important part in developing modern agriculture in China, and they bear the important responsibility of guaranteeing national grain security. Their agricultural population accounts for 80% of the total agricultural population and 60% of the total population in China. Their cultivated land area and grain sown area account for more than 60% of the national total, and their grain output accounts for 70% of the national total. However, these major grain-producing areas have encountered bottlenecks in grain yield increase. In particular, serious over-exploitation of groundwater, predatory management of farmland, and long-term extensive use of chemical fertilizers have led to the decline of the quality of cultivated land, the degradation of soil desertification, serious soil erosion, and water pollution. In short, grain production faces many new challenges in these major grain-producing areas.

In this research, we focus on rice, wheat and corn. We measure quality of rice, wheat, and corn using their first-class rates. Due to the lack of detailed data, for transportation (measured by road mileage), labor input, machinery use, fertilizer input, and fiscal support, data from different agricultural departments are used, and these data are also excerpted from the China Grain Yearbook and the website of the National Bureau of Statistics of China.

### 3.1. Analysis of Determinants of Grain Quality

The panel data of the 13 major grain producing areas in China from 2008 to 2015 are used, and based on the one-step stochastic frontier model, we presented the estimated result of determinant model of rice, wheat, and corn in Table 1. Models (1)–(3) present the determinant model of rice, wheat, and corn, respectively. In order to judge whether the stochastic frontier model is proper, the paper reports the *γ* statistics, assuming $\gamma =\sigma_{u}{}^{2}/(\sigma_{u}{}^{2}+\sigma^{2})$. γ represents proportion of inefficiency to stochastic disturbance of all models. If it is close to 1, it means that stochastic frontier model is proper, and vice versa. The *γ* values of all models in Table 1 approach 1. This means that the errors of leading-edge quality function mainly come from inefficiency. Hence, it is proper to estimate with stochastic frontier model.

According to the estimate result of the one-step stochastic frontier model, in the determinant of rice model (Model (1)), the estimated coefficient of transportation lnT is significantly positive. This shows that one of the important reasons for the grain quality improvement is the improvement of transportation. The coefficient of cross term tlnT of transportation and time is also significantly positive. This means that as the time goes by, the impact of transportation on grain quality improvement becomes larger. In addition, in Model (1), the coefficient of labor force lnL is obviously positive, but its cross term with time tlnL variable is insignificant. The coefficient lnK of agricultural machinery is obviously positive, and this is the same with its cross term with time tlnK. This means that with the transformation of grain yield pattern, the contribution of agricultural machinery to grain quality improvement becomes increasingly prominent. The modern farming technology represented by agricultural machinery is replacing the traditional manual farming, which has become a new driving factor of the grain quality improvement. The coefficient lnF of fertilizer use is obviously negative, and its cross term with time tlnF is insignificant. In addition, lnZ is positive, and tlnZ is insignificant. This shows that although the fertilizer and fiscal support are still important methods to influence grain quality, the effectiveness will go down as time goes on.

The results of Models (2) and (3) are similar to those of Model (1). Labor coefficient lnL is significant, tlnL is not significant, agricultural machinery coefficient lnK is significantly positive, and tlnK is significantly positive. This means that the use of modern farming technology represented by agricultural machinery has become a major factor of grain quality improvement. The fertilizer use coefficient lnF and fiscal support independent variable lnZ is significant, but their cross terms with time tlnF and tlnZ are insignificant. The coefficient of transportation lnT is positive in the model of wheat and corn, and its cross term with time tlnT is obvious positive. This is the same with the rice model.

### 3.2. Analysis of Grain Quality Improvement Potential and Efficiency

**Figure 2:**
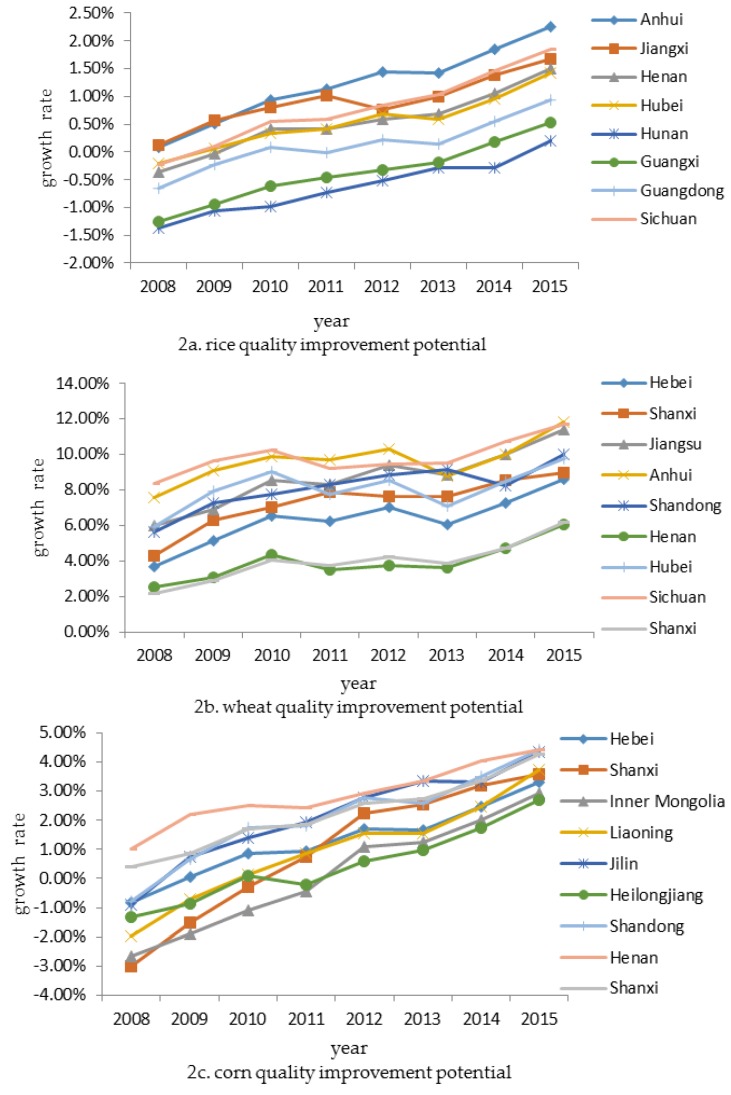
Trends of grain quality improvement potential in all samples.

Based on the panel data from 2008 to 2015 of the major grain producing areas and Equation (7), we measured the rice, wheat and corn quality improvement potential in sample provinces and regions. Figure 2 illustrates the trends of quality improvement potential, where the vertical axis represents growth rate of quality improvement potential. It shows that the growth rate of grain quality improvement potential in most of samples is positive, and the growth rate tends to increase steadily. Specifically, Figure 2a shows the growth rate of rice is low and less than 0.02 for most of those years. In contrast, Figure 2b shows the growth rate of wheat is high, and Figure 2c shows that the growth rate of corn is around 0.03 in 2015.

**Figure 3:**
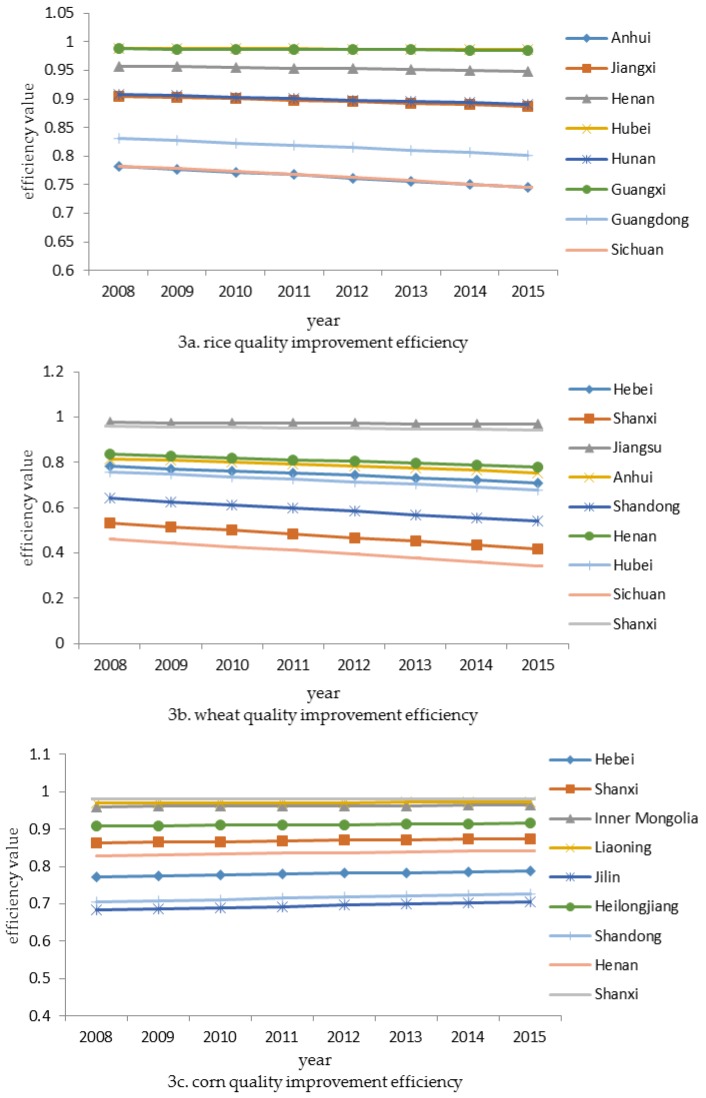
Trends of grain quality improvement efficiency in all samples.

Based on Equation (8), we plotted the changes in grain quality improvement efficiency in Figure 3. Figure 3a shows that the quality improvement efficiency of rice is relatively high, but it shows a downward trend. This is similar to wheat whose growth rate goes down even though a part of the provinces’ value is low as shown in Figure 3b. In contrast, Figure 3c shows that the quality improvement efficiency of corn goes up, but it increases slowly.

## 4. Empirical Research on the Relationship between the Grain Quality and Grain Yield

The previous empirical study found that China’s grain quality improvement potential was experiencing an upward trend, thus the improvement of China’s grain quality is sustainable. However, it is still interesting to see if the grain yield growth can also be sustainable. Therefore, we must further understand whether food quality improvement is beneficial or not to the growth of grain yield. To answer this question, we build the following model on how grain quality impacts grain yield:

(9) $$\ln Y_{it}=c+\alpha_{1}t+\alpha_{2}t^{2}+\psi \ln Q_{it}+\alpha_{3}\ln T_{it}+\alpha_{4}\ln L_{it}+\alpha_{5}\ln K_{it}+\alpha_{6}\ln F_{it}+\alpha_{7}\ln Z_{it}\;+\delta_{Y}t\ln Q_{it}+\delta_{D}t\ln T_{it}+\delta_{L}t\ln L_{it}+\delta_{K}t\ln K_{it}+\delta_{F}t\ln F_{it}+\delta_{Z}t\ln Z_{it}+V_{it}-u_{it}$$

(10) $$\ln Y_{it}=c+\alpha_{1}t+\alpha_{2}t^{2}+\psi \ln g_{it}+\alpha_{3}\ln T_{it}+\alpha_{4}\ln L_{it}+\alpha_{5}\ln K_{it}+\alpha_{6}\ln F_{it}+\alpha_{7}\ln Z_{it}\;+\delta_{g}t\ln g_{it}+\delta_{D}t\ln T_{it}+\delta_{L}t\ln L_{it}+\delta_{K}t\ln K_{it}+\delta_{F}t\ln F_{it}+\delta_{Z}t\ln Z_{it}+V_{it}-u_{it}$$

(11) $$\ln Y_{it}=c+\alpha_{1}t+\alpha_{2}t^{2}+\psi \ln TE_{it}+\alpha_{3}\ln T_{it}+\alpha_{4}\ln L_{it}+\alpha_{5}\ln K_{it}+\alpha_{6}\ln F_{it}+\alpha_{7}\ln Z_{it}\;+\delta_{TE}t\ln TE_{it}+\delta_{D}t\ln T_{it}+\delta_{L}t\ln L_{it}+\delta_{K}t\ln K_{it}+\delta_{F}t\ln F_{it}+\delta_{Z}t\ln Z_{it}+V_{it}-u_{it}$$

In Formulas (9)–(11), Y represents the grain yield. In Formula (9), Q represents the grain quality measured by the proportion of the first grade; g in Formula (10) and TE in Formula (11) represent grain quality improvement potential and the grain quality improvement efficiency. Considering the endogeneity problems posed by the bi-directional causal relationship between grain quality and grain yield, empirical tests of Models (9)–(11) use system GMM estimation of dynamic panel models. By referring to specific model estimation, lnQ, tlnQ, lng, tlng, lnTE, and tlnTE are regarded as endogenous variables, and other variables are regarded as exogenous.

Based on the one-step system GMM estimation method, Table 2 reports the estimation results of the corresponding equations. Models (4)–(6) in Table 2 are empirical estimates of rice yield as a dependent variable. Models (7)–(9) are empirical estimates of wheat yield as a dependent variable. Models (10)–(12) are the estimation of corn yield as the dependent variable. From the diagnostic tests of each model in Table 2, the selected tool variables and their lag orders are suitable. The AR (2) test shows that there is no second-order autocorrelation for the residuals obtained from the difference equation. Hansen test shows that the overidentification condition is verified.

In Models (4)–(6) of Table 2, the estimated coefficients of lnQ and tlnQ are not significant, indicating that the improvement of rice quality doesn’t have a significant impact on the yield. And the estimated coefficients lnTE and tlnTE are not significant, indicating that rice quality improvement efficiency will not affect its yield. The estimated coefficient lng is insignificant, indicating that the impact of rice quality improvement potential on rice yield is insignificant, while the estimated coefficient tlng is significantly positive. This result shows that rice quality improvement potential will significantly promote the yield increase. Therefore, rice quality improvement potential can not only sustain rice quality improvement, but also further improve the rice yield.

From Models (7)–(9), the estimation of wheat yield shows that the estimated coefficients of tlnQ, lnTE, tlnTE, and lng are insignificant, which is similar to our earlier empirical results. However, the estimated coefficient of lnQ is significantly negative while the estimated coefficient tlng is significantly positive. This shows that the traditional wheat production methods and wheat quality improvement are in conflict. With the change of time, the quality improvement potential can be expanded to achieve the dual objectives of wheat quality and yield increase at the same time. Further, we observe Models (10)–(12), which take the corn yield as the dependent variable. The results of the estimation showed that all of lnQ, tlnQ, lnTE, tlnTE, lng, and tlng fail to reach the 10% significant level, although the *t* statistic of the estimated coefficient tlng is 1.61, and it is close to 10% significant level. This shows that the promotion effect of quality improvement potential on corn yield can be further manifested.

In conclusion, the estimation results in Table 2 show that the quantity-oriented grain production and quality improvement are not compatible with each other, especially in wheat production. Only by expanding grain quality improvement potential can we simultaneously achieve the dual objectives of improving grain quality and increasing grain yield.

## 5. Conclusions

In this research, we collected panel data from China from 2008 to 2015 in 13 major grain producing provinces, including Hebei, Henan, Heilongjiang, Jilin, Liaoning, Hubei, Hunan, Jiangsu, Jiangxi, Inner Mongolia, Shandong, Sichuan, and Anhui. We employ the stochastic frontier analysis framework and find that the use of agricultural machinery and the improvement in transportation have become the major driving forces of grain quality improvement in China. On the other hand, we find that the effectiveness of traditional factors, such as labor, fertilizer, and fiscal support, has deteriorated. Employing the stochastic frontier model, we further measure the trend of potential of grain quality improvement in the sample provinces and regions. We find that the potential of grain quality improvement in all sample provinces and regions rose steadily. However, the quality improvement potential of rice improved more slowly than those of wheat and corn. In short, we can conclude that China’s grain quality improvement is indeed sustainable. In addition, we find that only by expanding grain quality improvement potential can we simultaneously achieve the dual objectives of improving grain quality and increasing yield.

## Figures and Tables

**Table 1:** Estimate of the determinant of the grain quality.

Explanatory Variable	Explained Variable: The Logarithm of Grain Quality		
	(1) Rice	(2) Wheat	(3) Corn
t	0.0212 (0.44)	0.2133 (4.15) ***	−0.1243 (−0.39)
$t^{2}$	0.0142 (2.17) ***	0.0157 (3.07) ***	0.0198 (1.81) *
lnT	0.5534 (6.15) ***	0.3325 (5.32) ***	0.1845 (7.21) ***
tlnT	0.0134 (3.14) ***	0.0102 (2.33) **	0.0134 (2.83) ***
lnL	0.1451 (1.82) ***	0.1126 (2.48) **	0.1455 (2.16) **
tlnL	0.0022 (1.47)	0.0036 (0.43)	0.0054 (0.88)
lnK	0.1523 (2.78) ***	0.2097 (3.15) ***	0.3512 (2.19) **
tlnK	0.0101 (2.14) **	0.0094 (0.61)	0.0173 (2.36) **
lnF	−0.1703 (0.17)	−0.1646 (1.17)	−0.2012 (1.70) *
tlnF	−0.0122 (−0.22)	−0.0335 (−2.13) **	−0.0163 (−2.89) ***
lnZ	0.3315 (3.40) ***	0.2667 (2.02) **	0.2678 (1.79) *
tlnZ	−0.0237 (−3.27) ***	−0.0257 (−2.13) **	−0.0162 (−2.06) **
Cons	−0.2144 (−1.77) *	−1.8454 (−3.01) ***	−4.4612 (−3.33) ***
*γ*	0.9654	0.9245	0.9022
*η*	−0.0469 (−0.77)	−0.0277 (−4.22) ***	0.0278 (0.33)
Logfunction value	150.7534	149.9766	152.9643
Number of observations	72 = 8 × 9	81 = 9 × 9	81 = 9 × 9

Note: *t* statistics in parenthesis. */**/*** denotes significance at the 10%/5%/1% level.

**Table 2:** Estimate of the grain quality influencing grain yield.

Explanatory Variable	Explained Variable: The Logarithm of Rice Yield			Explained Variable: The Logarithm of Wheat Yield			Explained Variable: The Logarithm of Wheat Yield		
	(4)	(5)	(6)	(7)	(8)	(9)	(10)	(11)	(12)
lnQ	−0.0041 (−0.25)			−0.0114 (−1.85) **			0.0100 (0.71)		
tlnQ	0.0013 (0.89)			0.0008 (0.56)			0.0026 (1.03)		
lng		0.0032 (1.08)			0.0105 (0.59)			0.0008 (0.59)	
tlng		0.0065 (2.11) **			0.0004 (3.07) ***			0.0017 (1.61)	
lnTE			0.0008 (0.22)			0.0014 (0.47)			0.0022 (0.34)
tlnTE			0.0010 (0.43)			0.0025 (1.00)			0.0005 (1.12)
AR(2)	0.225	0.562	0.567	0.579	0.278	0.845	0.526	0.136	0.526
Hansen test	0.277	1.000	0.566	1.000	0.577	0.628	0.589	0.979	0.478
Number of observations	72 = 8 × 9	72 = 8 × 9	72 = 8 × 9	81 = 9 × 9	81 = 9 × 9	81 = 9 × 9	81 = 9 × 9	81 = 9 × 9	81 = 9 × 9

Note: The model controls the first-order lag of dependent variables and other explanatory variables, t, $t^{2}$, lnT, tlnT, lnL, tlnL, lnK, tlnK, lnF, tlnF, lnZ, tlnZ in the equation, in order to save space, they are not reported here. *t* statistics in parenthesis. */**/*** denotes significance at the 10%/5%/1% level.
